# Assessing tumor contrast in radiographically dense breast tissue using Diffuse Optical Spectroscopic Imaging (DOSI)

**DOI:** 10.1186/bcr3485

**Published:** 2013-09-26

**Authors:** Anaïs Leproux, Amanda Durkin, Montana Compton, Albert E Cerussi, Enrico Gratton, Bruce J Tromberg

**Affiliations:** 1 Irvine, Beckman Laser Institute, University of California, 1002 Health Sciences Road, Irvine, CA 92612, USA; 2 Irvine, Laboratory for Fluorescence Dynamics, University of California, Irvine, CA 92612, USA

## Abstract

**Introduction:**

Radiographic density adversely affects the performance of X-ray mammography and can be particularly problematic in younger and high-risk women. Because of this limitation, there is significant ongoing effort to develop alternative cancer screening and detection strategies for this population. This pilot study evaluates the potential of Diffuse Optical Spectroscopic Imaging (DOSI) to image known tumors in dense breast tissue.

**Methods:**

We performed a retrospective analysis on 24 radiographically dense breast cancer subjects measured with DOSI over a four-year period (Breast Imaging Reporting and Data System - BI-RADS, category 3 and 4, average age = 39 ± 7.6, average maximum size 31 ± 17 mm). Two previously-described DOSI contrast functions, the tissue optical index (TOI) and the specific tumor component (STC), which are based upon the concentrations and spectral signatures of hemoglobin, water and lipids, respectively, were used to form 2D optical images of breast tumors.

**Results:**

Using TOI and STC, 21 out of 24 breast tumors were found to be statistically different from the surrounding highly vascularized dense tissue and to be distinguishable from the areolar region. For these patients, the tumor to normal contrast was 2.6 ± 1.2 (range 1.3 to 5.5) and 10.0 ± 7.5 (range 3.3 to 26.4) for TOI and STC, respectively. STC images were particularly useful in eliminating metabolic background from the retroareolar region which led to identification of two out of four retroareolar tumors.

**Conclusions:**

Using both the abundance and the disposition of the tissue chromophores recovered from the DOSI measurements, we were able to observe tumor contrast relative to dense breast tissue. These preliminary results suggest that DOSI spectral characterization strategies may provide new information content that could help imaging breast tumors in radiographically dense tissue and in particular in the areolar complex.

## Introduction

Tumors and glandular tissue have a similar dense appearance on mammography, making it difficult to distinguish metabolically active normal breast tissue from cancer. As a result, the performance of mammography in women with high breast density is poor [1,2]. While the overall sensitivity of mammography is about 75% and can be as high as about 90% in postmenopausal fatty breasts, it drastically drops to about 50% and 30% in women with heterogeneous and extremely dense breasts, respectively [3-5]. Younger and high-risk women who tend to have denser breasts are particularly impacted by this limitation. Given these difficulties, there are conflicting opinions regarding the use of mammography. The National Cancer Control Network (NCCN) recommends annual screening for women over 40, while the U.S. Preventative Services Task Force concluded that the risk of false positives and complications from biopsies is too high for screening mammography in pre- and peri-menopausal women, that is, up to age 50 [6]. Although the risk of breast cancer is low in this population, women in their 40s account for at least a quarter of breast cancer diagnoses and up to 17% of breast cancer deaths each year [6]. The National Cancer Institute estimates that up to 20% of all breast cancers, roughly 40,000/year in the U.S., are not discovered by screening mammography. Moreover, breast cancer at a young age tends to be more aggressive and less likely to respond to treatment as compared to breast cancer in older women [7]. This stresses the importance of early detection of breast cancer in women with dense breast tissue.

Detection and characterization of tumors in dense breasts have been investigated using several alternative approaches, such as magnetic resonance imaging (MRI), ultrasonography (UN), digital mammography and positron emission tomography (PET) [4,8-14]. Digital mammography has been shown to have better tumor detectability in dense breasts [13] compared to older film-based systems. The overall detection rate, however, is still moderate in younger and high-risk subjects [4]. Previous studies have shown that adding screening ultrasound to mammography can improve overall performance in dense breasts [4,8,11], and that breast MRI is generally not limited by breast density [12]. However, there are concerns that the use of both MRI and US may increase false positive rates. Breast-dedicated PET has been shown not to be independent of breast density and to have a better specificity than MRI [14]. However, PET relies on the injection of a radioactive tracer in the patient’s body.

Optical imaging has been predicted to improve sensitivity and specificity in dense breast tissues, but this has not yet been well established. Most published optical studies focusing on lesion detection and characterization were performed in heterogeneous breast density populations [15-21]. Increased hemoglobin and water content, as well as decreased lipid content were observed in tumors compared to normal surrounding breast tissue. Most studies have shown that tumor contrast in hemoglobin concentration is the strongest detection marker of tumors [22]. Mammographically dense breast tissue also contains higher hemoglobin content, as a result of the increased metabolic activity of fibro-glandular tissues with respect to fatty breast tissue. This can minimize the tumor to normal hemoglobin contrast limiting the effectiveness of Diffuse Optical Spectroscopic Imaging (DOSI) and related optical imaging techniques to identify tumors in dense breasts. There are few optical imaging studies that have examined mammographically dense breast tissue. For example, Taroni *et al*. proposed an optical index that correlates with mammographic density [23]. This index is derived from water, collagen and lipid content, combined with a tissue scattering parameter, and is measured using time-resolved transmittance spectroscopy. Blackmore *et al*. and Blyschak *et al*. investigated the correlation between mammographic density and optical transillumination spectroscopy [24,25]. The objective of these studies was to assess breast density as an indicator for breast cancer risk. To our knowledge, the visualization, detection or characterization of tumors in dense breast tissue has never been investigated using optical techniques.

In this paper, we present a pilot study that prospectively assesses the potential of DOSI to image breast cancer in 24 patients with dense parenchymal tissue of Breast Imaging Reporting and Data System (BI-RADS) 3 and 4 using two relatively new contrast functions. DOSI measures breast tissue physiological properties and composition (for example, tissue concentration of oxy- and deoxyhemoglobin, water and lipid). The DOSI method employed in this study is based on mapping tissue absorption and scattering spectra (650 to 1,000 nm) [26] acquired using a hand-held probe scanned over approximately 50 to 100 discrete locations on the breast for each subject. A tissue optical index (TOI) that is sensitive to the metabolic activity of breast tissue can be derived from these physiological properties [17]. Moreover, using a double differential spectroscopy technique, signatures of specific tumor components (STC) can be obtained. The STC is based on detecting small spectral shifts that are hypothesized to be related to the unique disposition of chromophores present in malignant tumors but not in normal or benign tissues [27]. Our results suggest that there is complementary information from TOI and STC regarding tissue breast tissue metabolism and composition. Together, these contrast functions were able to significantly discriminate 21 out of 24 breast tumors from highly vascularized dense tissue. These preliminary findings support the idea that DOSI spectral characterization strategies may provide new information content that could potentially support breast cancer detection in radiographically dense tissue.

## Methods

### DOSI instrument

A description of the basic components of our DOSI instrument has been previously presented [28,29]. Briefly, DOSI consists of a combined frequency-domain photon migration (FDPM) component and a broadband steady-state (SS) component integrated together to produce broadband absorption and scattering spectra of tissues from 650 to 1,000 nm. The FDPM component uses six laser diodes (Blue Sky Research, Sanyo, Mitsubishi, Japan) at the wavelengths 658, 682, 785, 810, 830 and 850 nm. The breast is illuminated sequentially by each laser diode, which is intensity-modulated at 401 modulation frequencies swept from 50 to 600 MHz. The back-scattered light is detected by an avalanche photodiode (Hamamatsu model C5658 module, customized with S6045-03 APD, Hamamatsu Photonics K.K., Solid State Division, 1126-1 Ichino-cho, Higashi-ku, Hamamatsu City 435-8558, JAPAN) mounted inside a hand-held probe. The SS component uses a high-intensity tungsten-halogen source (Mikropack model HL-2000-HP-FHSA, Ocean Optics, Inc., 830 Douglas Ave, Dunedin, FL 34698, USA) to illuminate the tissue and the back-scattered light is detected by a grating-based spectrometer (650 to 1,000 nm, 1,024 pixels, BWTek model 611E, B&W Tek, Inc., 19 Shea Way, Newark, DE 19713, USA). The FDPM and SS sources are coupled by optical fibers mounted into the hand-held probe. The separation between source and detector fibers was 28 mm for both FDPM and SS with fibers placed in an overlapping geometry [17].

FDPM and SS data are combined to provide broadband absorption (μ_a_) and reduced scattering (μs’) spectra from 650 to 1,000 nm. In the FDPM measurement, the phase and amplitude of the remitted light are recorded as functions of modulation frequency and fit to a diffusive model of light transport (semi-infinite boundary conditions) to recover μ_a_ and μs’ at each of the six laser wavelengths. SS broadband spectra were converted into absolute absorption spectra using two simple steps. First, the spectral shape of the reduced scattering spectrum is assumed to follow a power law of the form μs’ = Aμ^-sp^, where A is the scatter amplitude and sp is the scatter power, or the exponent of the scattering spectrum. The power-law fit to the FDPM discrete laser diode spectrum provides a scatter correction for the SS reflectance spectrum. We then fit the SS reflectance intensity at each of the laser diode wavelengths to the reflectance calculated from the FDPM-measured absolute absorption values. Thus, the SS reflectance spectrum intensity is scaled using the FDPM discrete laser diode measurements. The absolute absorption spectrum is then extracted by fitting the corrected reflectance spectrum to a diffusion reflectance model [29,30].

### Spectral analysis

Spectral analysis is performed on the absorption data assuming that the absorption in normal breast is caused mainly by the tissue concentration (ct) of four main chromophores: deoxyhemoglobin (ctHHb), oxyhemoglobin (ctO_2_Hb), water (ctH_2_O) and bulk lipid. We recover these chromophore concentrations by fitting a linear combination of their basis molecular extinction coefficient spectra to the scatter-corrected absorption spectrum [31]. A TOI representative of tissue metabolism has been introduced previously [17]: TOI = ctHHb x ctH_2_O/lipid. The TOI is typically used to identify the functional/spatial extent of the lesion [17,32] and areola.

The STC is determined by taking the difference between sample (that is, tumor) and reference (that is, healthy) tissue absorption spectra and analyzing the residuals of the fit of this first differential to the four-component basis chromophores of breast tissue. Details of this method have been reported [33,34]. Briefly, the first differential (between tumor and healthy tissue absorption spectra tissue) provides a spectrum that removes spectral components common to both tissues. The second differential (between the aforementioned first differential and its fit to the four-chromophore basis spectra) provides a spectrum containing the additional contributions of chromophores not accounted in this fit. This second differential fit is not to be confused with “second derivative spectroscopy” where derivatives of the measured intensity are fit to derivatives of the basis chromophore spectra to obtain concentrations of the basis components. Rather, localized subtle spectral features not described by the basis spectra are detected by fitting differences in absorption to the basis spectra of oxy- and deoxyhemoglobin, water and lipid in one molecular state. Thus, the residual of the chromophore fit to the first differential, known as the STC spectrum, emphasizes the contributions of other chromophores not accounted in the basis spectra fit (for example, met-hemoglobin), and contains information on shifts of water and lipid peaks that correlate with different molecular states that differ between the sample and reference locations.

In order to quantify the STC spectrum, an STC index was defined by the sum of all local residual variances L_k_ calculated over five specific spectral regions [33]:

(1) $$\text{STCindex}=\sum_{k=1}^{5}L_{k}=\sum_{k=1}^{5}\left(\sum_{i}\mathrm{ST}C_{i}{\left(\lambda_{i},x,y\right)}^{2}/N_{k}\right)$$

The local variance L_k_ is a function of the position on the breast given by x and y coordinates. The index k indicates a given spectral region and N_k_ indicates the total number of wavelengths in the spectral region. STC_i_(λ_i_, x, y) is the value of the STC spectra at a given wavelength. The spectral regions are as follows: 650 to 665 nm, 730 to 800 nm, 875 to 930 nm, 930 to 960 nm and 980 to 990 nm. These five regions have been defined empirically to maximize the differences between tumor and normal [33].

Using the TOI and STC index for each spatial measurement point of the breast (see below for measurement description), TOI and STC index maps can be obtained [34].

### Breast density

Breast density is often expressed using the BI-RADS. The BI-RADS classification system defines four categories for breast density, qualitatively based on the relative amounts of fat and dense fibroglandular tissue observed in a mammogram. BI-RADS 1 refers to almost entirely fatty tissue; BI-RADS 2 refers to scattered fibroglandular densities that could potentially obscure a lesion; BI-RADS 3 refers to a heterogeneously dense breast type and the sensitivity of mammography may be lowered; BI-RADS 4 refers to an extremely dense breast type that will lower the sensitivity of mammography [1].

### Subject selection and measurement procedure

A retrospective analysis was conducted on 24 breast cancer subjects (average age = 39 ± 7.6, range 25 to 50) who were measured with DOSI between 2007 and 2011 and met the criteria of breast tumors and dense breast tissue BI-RADS 3 and 4. Subject information is shown in Additional file 1. Lesion pathology was determined by core biopsy: the study included 17 invasive ductal carcinomas (IDC), 5 invasive lobular carcinomas (ILC) and 2 ductal carcinomas *in situ* (DCIS). Breast density was determined from mammography: 16 subjects had extremely dense breast tissue (BI-RADS 4), 7 subjects had heterogeneously dense breast tissue (BI-RADS 3). The breast density information was not available for one subject: this subject was included in the study as her tumor was retroareolar and was not visible in mammography. Out of these 24 subjects, 21 were premenopausal, 2 were perimenopausal and 1 was postmenopausal. The lesion sizes and locations were determined by ultrasound examination and ranged from 8 to 70 mm with an average maximum size of 31 ± 17 mm. All subjects provided informed written consent under protocols approved by Institutional Review Board of the University of California, Irvine.

DOSI measurements were performed using a standard protocol [32]. The normal contralateral breast of five subjects was not measured with DOSI, though ipsilateral normal tissue was measured for all subjects. All subjects were measured in a supine position. The DOSI probe was placed against the breast tissue, and sequential measurements were taken in a rectangular grid pattern using 10-mm spacing. Broadband absorption and reduced scattering spectra (650 to 1,000 nm) were measured at each location. The dimension of the grids ranged from 3 x 9 to 10 x 9 cm^2^ for the normal contralateral breast and from 5 x 9 to 13 x 13 cm^2^ for the ipsilateral breast. For better visualization, the DOSI images presented in this paper are interpolated images.

### Data analysis

The areola was included, partly or entirely, in the field of view of the DOSI images in 20 out of the 24 subjects. For data analysis, the areolar regions were defined in the TOI images and on the actual physical size of the areola. The tumor region was defined around the peak values in the TOI and STC index images and using the tumor size given by the ultrasound examination. The normal tissue in the ipsilateral breast was defined as the field of view excluding the tumor and areola regions and an additional 1 cm wide zone around these regions. The tumor to normal (T/N) contrast was defined by the average value in the tumor region divided by the average value in the background normal tissue region [35].

In the box plots presented in this paper, the box represents the standard deviation and median of the depicted values, the bar represents the total range (minimum to maximum), and the square represents the average value.

In the DOSI images, we refer to as “Cancelled Areolas” the areola regions for which the DOSI signal is found statistically lower than the signal from the tumor. Visually, this corresponds to a highly attenuated signal at the areola regions in the DOSI image due to the STC spectral processing. Otherwise, we refer to the cases as “Not Cancelled Areola”. We expect better identification of tumors in the cases of “Cancelled Areola” as the areolar signal does not obscure the signal from the tumor.

### Consent section

Written informed consent was obtained from the patients for publication of this manuscript and accompanying images. Copies of the written consents are available for review by the Editor-in-Chief of this journal.

### Statistical analysis

Statistical significance between the two groups was performed using a one-tailed Wilcoxon test at alpha = 0.05.

The statistical significance between tumor and normal dense tissue was established in the TOI and STC index images of each patient in several steps. First, the statistical significance of the difference between tumor and normal tissue, as defined in the Data analysis section, was assessed for both the STC and TOI datasets of each patient. Then, for the subset of STC and TOI data exhibiting statistical significance, we rejected the cases of missed tumors, which we defined as cases where the signal from the normal tissue was greater than from tumor tissue. For this resulting subset of data, we determined the statistical significance between tumor and areola regions in the case of areolar tumors. Two areolar tumors that were not visually separated from the areola were also excluded from the analysis. The number of STC and TOI datasets exhibiting statistical difference between tumor and normal dense breast tissue were based upon the final resulting datasets of this process.

## Results

**Figure 1:**
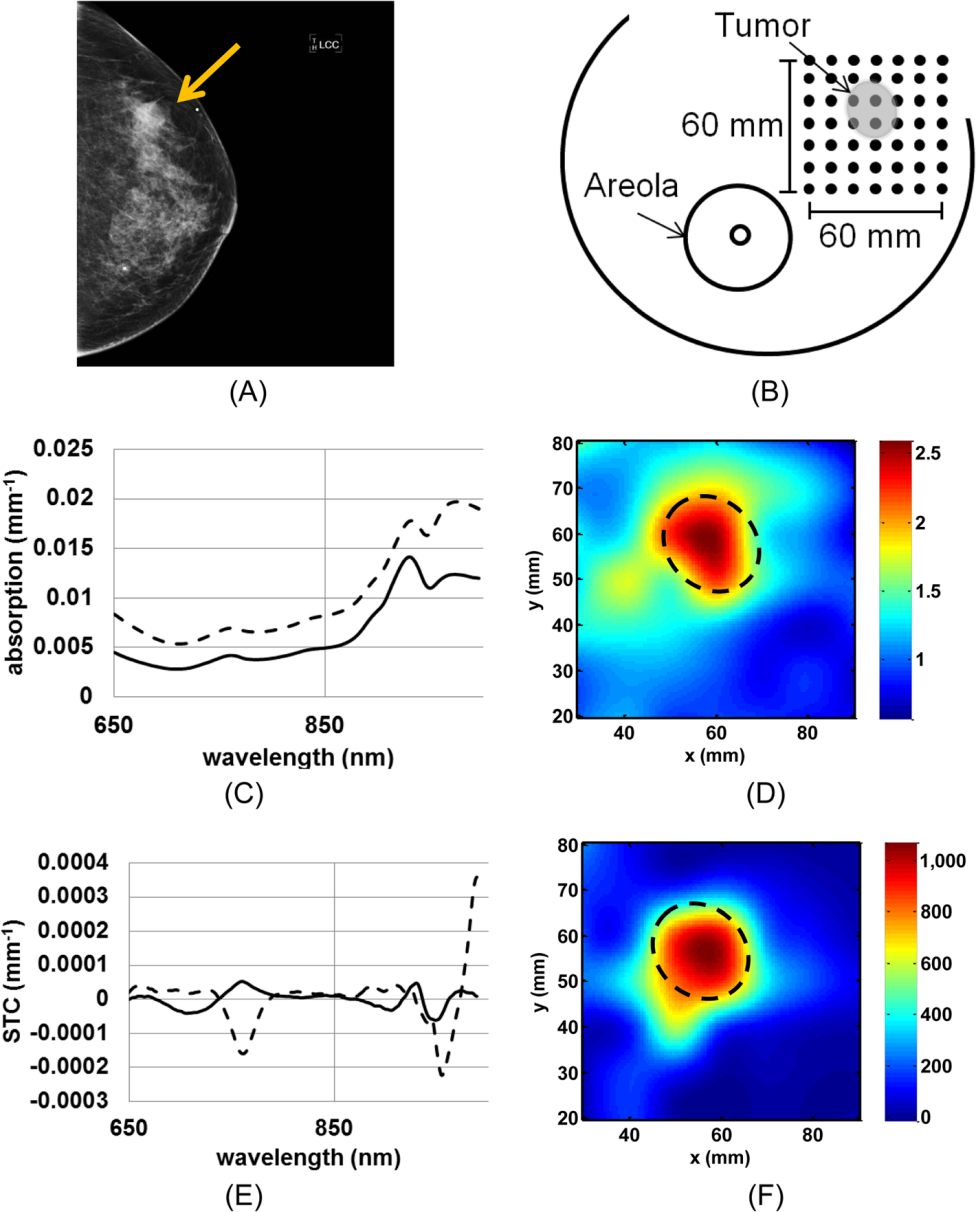
**DOSI measurement with typical recovered absorption and STC spectra in tumor and normal tissue. A)** Mammogram, left craniocaudal view, of a 39-year-old female subject with a 21 mm IDC in the left breast with a BI-RADS 3 density. The arrow points at the tumor. **B)** Schematic of the DOSI measurement grid for this subject. DOSI measurement locations are 10 mm apart in x and y directions. For this subject, the 60 x 60 mm^2^ grid covers the lesion and some normal tissue. **C)** Absorption spectra in lesion (dotted line), and background (solid line). **D)** TOI map. **E)** STC spectra in lesion (dotted line), and background (solid line). **F)** STC index map. Increased TOI and STC index values are found in the tumor area, highlighted with the dash line circle. BI-RADS, Breast Imaging Reporting and Data System; DOSI, Diffuse Optical Spectroscopic Imaging; IDC, Invasive ductal carcinomas; STC, Specific tumor component; TOI, Tissue optical index.

We depict in Figure 1 the DOSI measurement process of patient #3, a 39-year-old premenopausal female with a 21 mm IDC in the upper outer part of her left breast. Figure 1A presents the left craniocaudal view of the mammogram of this patient. She was assessed with BI-RADS 3 mammographic density. The arrow points at the tumor location. Figure 1B illustrates the 60 x 60 mm^2^ DOSI measurement grid performed on this patient relative to the tumor position and size, and areola. Figure 1C shows the scatter-corrected absorption spectra of a tumor location (dotted line) and a normal healthy tissue location (solid line) of this breast. The lesion has a substantially higher absorption than the normal breast tissue with elevated hemoglobin (mainly 650 to 850 nm) and water (mainly 950 to 1,000 nm) [17]. Figure 1D presents the resulting TOI image of this measurement; note that this image is not normalized. An area with increased TOI value is observed and corresponds to the tumor location known from ultrasound. Figure 1E presents the STC spectra of the same tumor and normal tissue locations as shown in Figure 1C. The tumor STC spectrum (dashed line) contains greater peaks in absolute value in the hemoglobin region and in the water region as compared to the normal tissue STC spectrum (solid line) [27]. This can be interpreted as the tumor region having greater malignant components than the surrounding healthy tissue. Figure 1F shows the corresponding STC index image of this measurement. An increased STC index value is observed in the tumor region [36]. Note that the TOI and STC index maps are top-down views.

**Figure 2:**
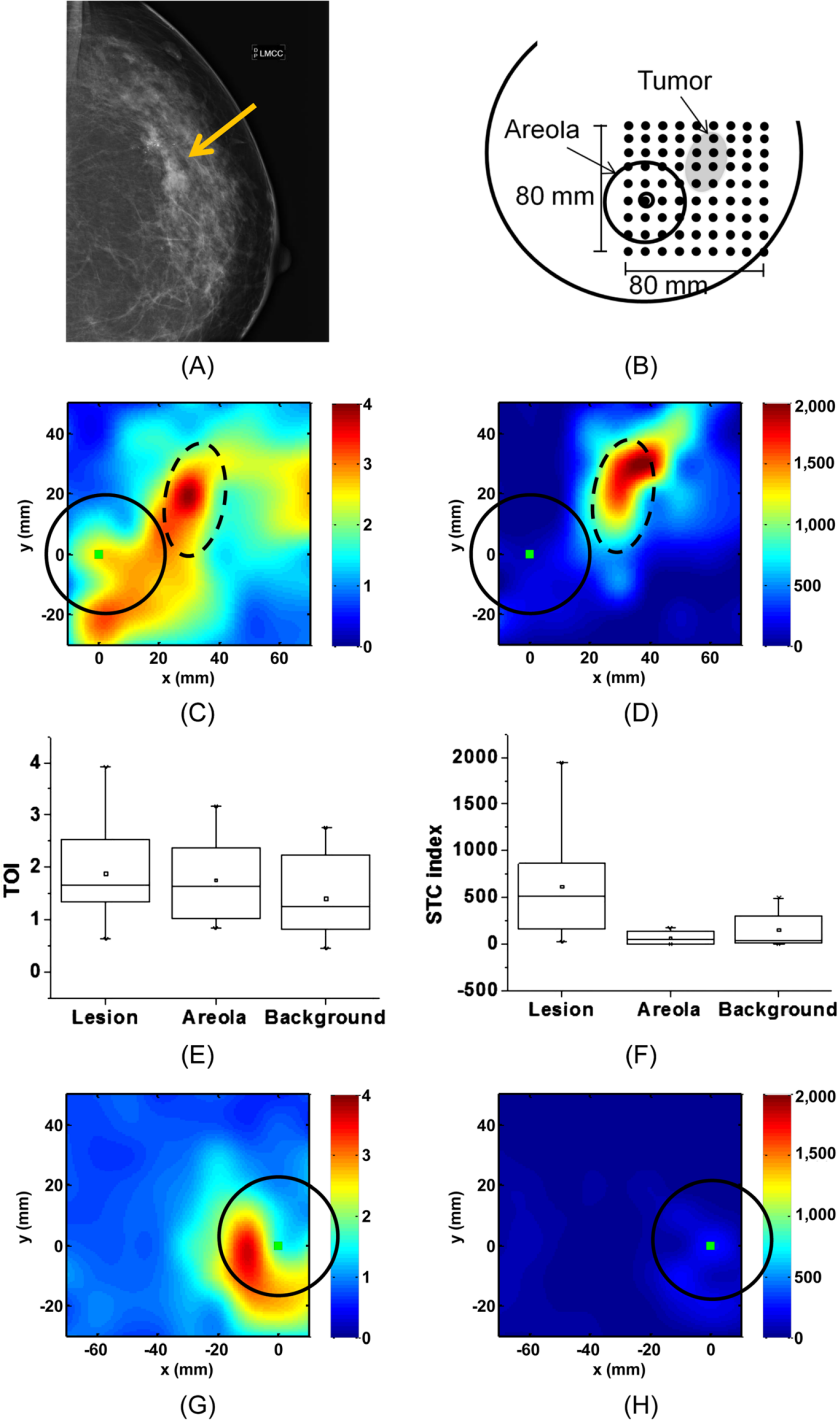
**DOSI results of subject #5, IDC in the left breast, breast density BI-RADS 4. (A)** Mammogram, left medio-craniocaudal view. The arrow highlights the tumor location. **(B)** Schematic representation of the DOSI measurement in the ipsilateral breast. **(C)** TOI map of the ipsilateral breast. **(D)** STC index map of the ipsilateral breast. **(E)** TOI values in tumor, areola and normal tissue of the ipsilateral breast. **(F)** STC index values in tumor, areola and normal tissue of the ipsilateral breast. **(G)** TOI map of the contralateral breast. **(H)** STC index map of the contralateral breast. The square represents the nipple location. The areolas are highlighted with the solid line circle, and the tumor by the dash line circle. BI-RADS, Breast Imaging Reporting and Data System; DOSI, Diffuse Optical Spectroscopic Imaging; STC, Specific tumor component; TOI, Tissue optical index.

Figure 2 illustrates a challenging case confounded by the dense areola tissue. The subject is a 49-year-old premenopausal female with IDC in the upper outer quadrant of the left breast and with a breast density of BI-RADS 4 (Patient #5). Figure 2A shows the mammogram of the ipsilateral breast of this subject in the medio-craniocaudal view. Figure 2B shows the schematic representation of the DOSI measurement. Figure 2C, D presents the TOI and STC index images of the ipsilateral breast, respectively, and Figure 2G, H presents the TOI and STC index images of the normal breast, respectively. In Figure 2C, we observe a global increase in TOI value in the whole field of view. However, the tumor (dashed line) shows the highest TOI values. The TOI variations observed around the tumor and areola (solid line) regions are most probably due to normal physiology of this highly dense breast tissue. Because the enhancements from the tumor and glandular tissue are connected, this tumor is difficult to observe in the TOI image. In Figure 2D, we observe an increase in STC index value mainly in the tumor region. TOI fluctuations are also present in the background but are negligible compared to the signal from the lesion. Note that there is insufficient contrast in the STC index map to reveal the areola. Figure 2E, F show the box plots of the TOI and STC index values of tumor, areola and normal tissue in the ipsilateral breast. As visually observed in Figure 2C, the highest TOI values are found in the tumor, but the areola and normal background tissue also exhibit high TOI values. Figure 2F shows that the STC signal from the tumor is dominant over the normal dense breast tissue including the areola, as visually observed in Figure 2D. Figure 2G, H shows the TOI and STC index maps, respectively, of the contralateral breast of this subject. The areola region and glandular tissue are again mainly observed in the TOI image.

**Figure 3:**
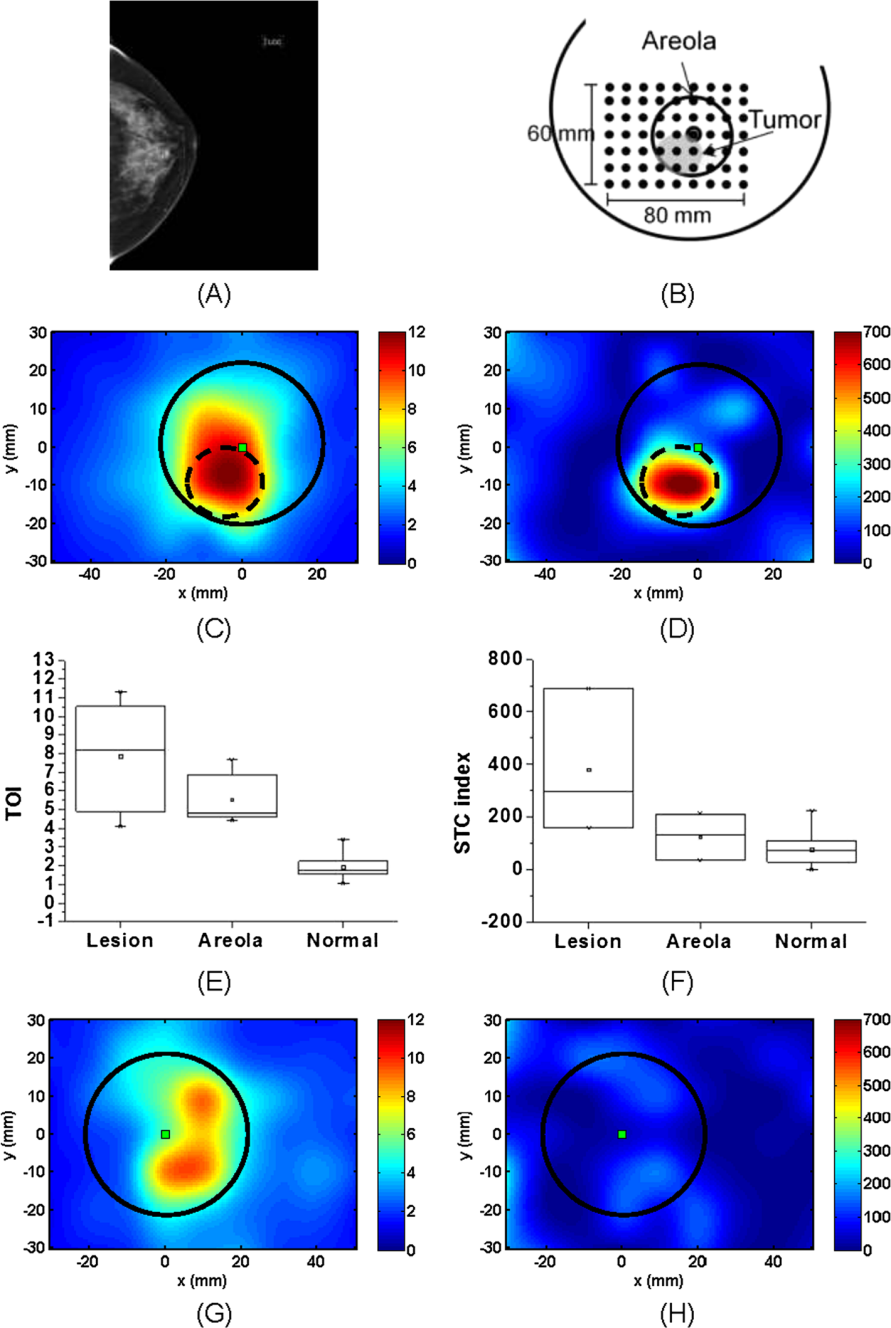
**DOSI results of subject #17, a 42 y.o. woman with a retroareolar 23 mm IDC. (A)** Mammogram, left craniocaudal view. **(B)** Schematic representation of the DOSI measurement. **(C)** TOI map of the ipsilateral breast. **(D)** STC index map of the ipsilateral breast. **(E)** TOI values in tumor, areola and normal tissue of the ipsilateral breast. **(F)** STC index values in tumor, areola and normal tissue of the ipsilateral breast. **(G)** TOI map of the contralateral breast. **(H)** STC index map of the contralateral breast. The square represents the nipple location. The areolas are highlighted with the solid line circle, and the tumor by the dash line circle. IDC, Invasive ductal carcinomas; STC, Specific tumor component; TOI, Tissue optical index.

We present in Figure 3 a more challenging case of a retroaerolar lesion not detected by mammography. This subject (patient #17) is a 42-year-old postmenopausal female with a 23 mm retroareolar IDC at 6 to 7 o’clock in the right breast. Figure 3A presents the mammogram of this breast in the craniocaudal view, and Figure 3B shows the DOSI measurement configuration. In Figure 3C, we observe a general increase of TOI in an extended region of the areola (the boundary of the areola is depicted by the solid line). The lesion, highlighted by the dashed line, cannot reliably be discriminated from the areola in this TOI map. In Figure 3D, we observe an increase in STC index value in the tumor region, which is overlaid by the areola region. Figure 3E, F presents the box plots of the TOI and STC index values of tumor, areola and normal tissue in the ipsilateral breast. In these plots, the areola region excludes the area of the tumor. In Figure 3E, the peak TOI value is observed at the tumor location, but both the areola and the tumor have similar TOI values, and the areola and tumor cannot be spatially separated. The plot in Figure 3F illustrates that the STC signal from the tumor is different from the STC signal from the areola and normal background tissue. This difference is statistically significant (Wilcoxon test at alpha = 0.05, *P*-value <0.01). Figure 3F also shows that the STC values from background and areola range similarly, implying that the STC contrast function may be insensitive to breast tissue density for this patient [34]. Figure 3G, H shows the TOI and STC index maps, respectively, of the contralateral breast of this subject. The areola, contained within the solid line, is observed in the TOI map but has a very low STC index signal. The contralateral STC index maps show little enhancement from the areola, suggesting that the increased values observed in the ipsilateral STC index are mainly due to the tumor.

**Figure 4:**
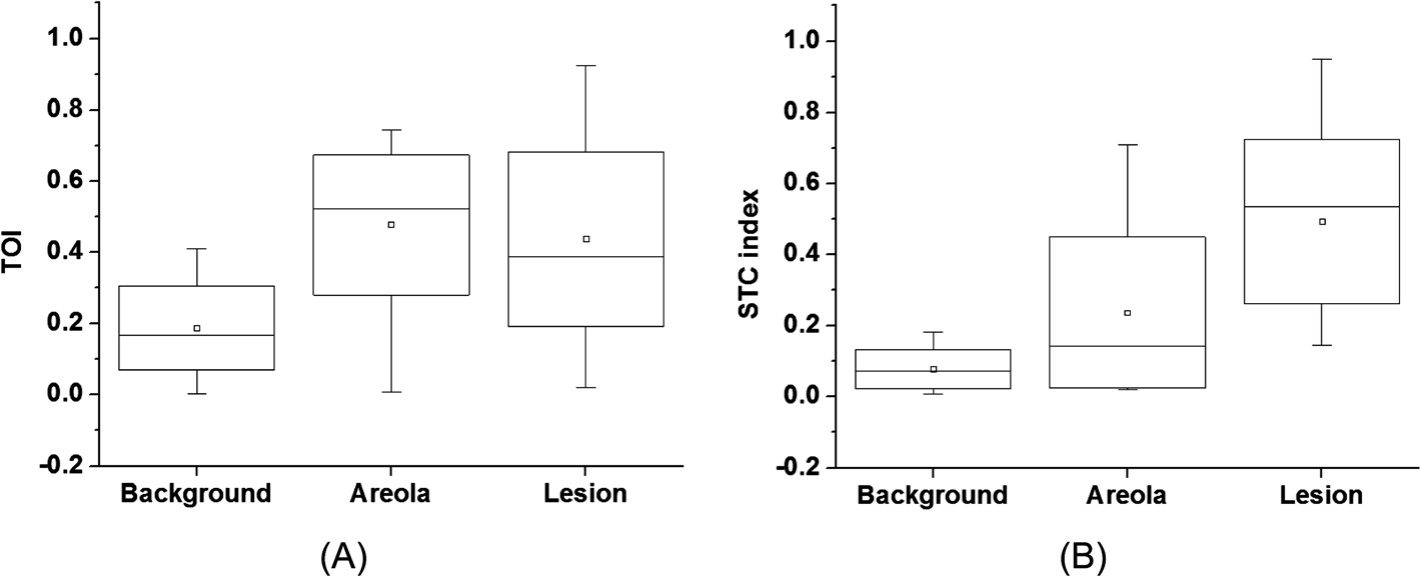
**Box plot of the TOI and STC index in tumor, areola and normal tissue. (A)** TOI values in the visible tumor, areola and normal tissue of the ipsilateral breast. **(B)** STC index values in the visible tumor, areola and normal tissue of the ipsilateral breast. STC, Specific tumor component; TOI, Tissue optical index.

Table 1 reports the average (standard deviation and range) of the mean ctO_2_Hb, ctHHb, H_2_O, lipid and TOI concentrations in the tumor, normal healthy breast tissue, and areolar tissue. Elevated blood, water and TOI as well as decreased lipid content are on average observed in tumors and areolas compared to normal dense breast tissue. The mean DOSI parameter differences between tumor and normal dense tissue are all statistically significant (Wilcoxon test at alpha = 0.05, *P* <0.01), while only oxyhemoglobin is statistically different between tumor and areolar tissue (Wilcoxon test at alpha = 0.05, *P* <0.01). Figure 4 presents the normalized TOI and STC index values for the 24 subjects in tumor, background and areola. We observe that the background signal from dense breast tissue is on average lower in the STC index images than in the TOI images.

**Table 1:** Average and standard deviation (minimum to maximum) of the mean chromophore values in tumor and normal breast tissue

	**Tumor**	**Normal**	**Areola**
ctO_2_Hb	26.8* ± 10.7	17.8 ± 4.9	20.1** ± 5.9
	(9.9 to 59.1)	(9.7 to 32.9)	(10.7 to 33.2)
ctHHb	8.3* ± 2.1	5.4 ± 1.2	8.1† ± 3.0
	(4.1 to 11.8)	(3.7 to 8.5)	(4.8 to 17.0)
H_2_O	37.9* ± 12.9	26.9 ± 10.3	45.4† ± 11.7
	(17.7 to 66.5)	(17.0 to 64.3)	(25.1 to 76.6)
lipid	58.2* ± 11.3	65.9 ± 11.3	51.6† ± 10.6
	(35.4 to 88.6)	(32.4 to 95.4)	(28.6 to 71.9)
TOI	6.8* ± 5.5	2.9 ± 3.4	9.5† ± 8.2
	(1.3 to 25.1)	(0.9 to 18.1)	(1.8 to 38.0)
STC	1,968.9* ± 3,701.0	336.2 ± 900.2	935.1 ± 1,688.3
	(21.3 to 16,509.9)	(16.1 to 4,450.0)	(21.9 to 7,085.9)

*, **, † denote a statistically significant difference (Wilcoxon test at alpha = 0.05, *P* <0.01) between tumor and normal, between tumor and areola, and between areola and normal, respectively. ctO_2_Hb, Concentration of oxy-hemoglobin (μM); ctHHB, Concentration of deoxyhemoglobin (μM); STC, Specific tumor component; TOI, Tissue optical index.

Statistically, 20 out of 24 lesions were significantly different from the background normal tissue in the TOI images (Wilcoxon test at alpha = 0.05, *P*-value <0.04) and 22 out of 24 lesions in the STC index images (Wilcoxon test at alpha = 0.05, *P*-value <0.04). For four subjects, the signal from the tumor was statistically different either in the TOI image or in the STC index image. Only one lesion was found not statistically different from the background in both the TOI and STC index images. This lesion is a 16 mm DCIS tumor in a 25-year-old subject with breast density of BI-RADS 4. An enhancement at the tumor location was nevertheless observed in the TOI image with a contrast of 1.3.

Unrelated to the statistical difference between tumor and background normal tissue, the tumor region could not be visually spatially separated from the areolar region in six patients in the TOI image and in two patients in the STC index image due to the extended presence of glandular tissue. For the tumors statistically different from normal dense breast tissue and spatially indistinguishable from the areola, the mean T/N contrast was 2.6 ± 1.2 (range 1.3 to 5.5) and 10.0 ± 7.5 (range 3.3 to 26.4) in the TOI and STC index maps, respectively.

Among the four areolar lesions, two were not discriminated from the areola in both the TOI and STC index images. In one subject, the lesion was separated from the areola in both the TOI and STC index images. In the last subject, shown in Figure 3, the lesion could not be distinguished from the areola in the TOI image, but was clearly resolved from the areola in the STC index image. We observed an attenuated STC signal from the glandular tissue including the areolar region in 11 subjects out of the 20 with the areola in the field of view, as seen for instance in patients presented in Figures 2 and 3.

## Discussion

Using the TOI and STC index, 21 out of 24 breast tumors were found to be statistically different from the surrounding normal dense breast tissue and to be distinguishable from the areolar region. Two out of the four areolar lesions were discriminated from the areolar region in the optical images. Drastic attenuation of the STC signal from the areola and dense breast tissue was observed in 11 out of the 20 subjects who had the areola in the field of view.

Dense breast is characterized by a relatively high fibro-glandular-to-lipid tissue ratio compared to normal breast. The tissue chromophore concentrations presented in Table 1 support this observation and agree with O’Sullivan *et al.* who reported a significant difference in deoxyhemoglobin, TOI and water between pre- and post-menopausal patients [37]. These changes reflect the greater vascular density, water content and metabolic activity of dense breast tissue [38]. The elevated mean deoxyhemoglobin and water content found in highly glandular areolar tissue, (Table 1), is also consistent with this concept.

A total of 16 out of 24 tumors were statistically different from the background dense breast tissue and could be distinguished from the areola region in the TOI map only. For these 16 tumors, the TOI T/N contrast was 2.6 ± 1.2 (range 1.3 to 5.5). For comparison, the areola to normal contrast in TOI was 3.1 ± 2.3 (range 1.1 to 8.4). Although TOI contrast may exist between tumor and glandular tissue, a high TOI signal is not exclusive to tumor and can be observed in fibroglandular tissue. Significant increases in mean hemoglobin, water and TOI, and decreases in mean lipids were observed in tumors relative to normal breast tissue, see Table 1. These differences are a direct result of tumor physiology and metabolism, including angiogenesis, cellular proliferation (often very high in young patients) and edema [15,17,39]. However, mean deoxyhemoglobin, water and lipid content was comparable for tumor and areola regions. These results suggest that even though contrast in TOI should be observable between tumor and glandular tissue, the highly-dense areolar complex may impact tumor to normal contrast. This explains why only 16 out of the 24 tumors were distinguishable from the normal dense breast tissue in the TOI images.

The STC index quantifies small spectral shifts present in the NIR absorption spectra of lesions that are believed to be related to the unique molecular disposition of chromophores present in malignant tumors [40,41]. STC tumor to normal contrast is based upon the extent to which the different molecular environment in tumor versus healthy tissue is able to induce distortions in pure component spectra [42,43]. As a result, the STC has specificity for malignant tumor tissue and not for normal glandular/fibroglandular structures. The STC index is thus less sensitive to breast density than the TOI. This explains why STC values for 20 (versus 16 for the TOI) out of 24 tumors were statistically different from normal dense breast tissue and why STC index maps could be used to identify tumors in the areolar region. The STC index T/N contrast was 10.0 ± 7.2 (range 3.3 to 26.4), 3.8-fold higher than TOI T/N values. For comparison, the STC areola to normal contrast was 7.4 ± 17.9 (range 0.3 to 87.3). Even though the absolute contrast in areola is on average higher in the STC index images than in the TOI images (7.4 versus 3.1, respectively), the mean values in the areola relative to the tumor are lower in the STC index images than in the TOI images (0.4 ± 0.2 and 0.2 ± 0.2, respectively; see Figure 4). We also observe in Figure 4 that the background signal from the dense normal breast tissue in the STC index images is on average lower than the background normal signal in the TOI images (0.17 ± 0.10 and 0.06 ± 0.05, respectively). Figure 3 presents the case of an areolar tumor that was not detected using mammography. Even though TOI tumor to background contrast is observed at the tumor region, the lesion cannot be discriminated from the areola in the TOI image. The featureless STC index map of the opposite normal breast suggests that the STC is not sensitive to the areolar signal of this subject. As a result, we can assume that the STC index enhancement observed in the tumor breast (and, more specifically, at the tumor area as predicted by ultrasound (US)) is primarily due to the tumor. Similar attenuation of the areola region occurred in 11 out of 20 subjects and did not correlate with BI-RADS breast density.

**Figure 5:**
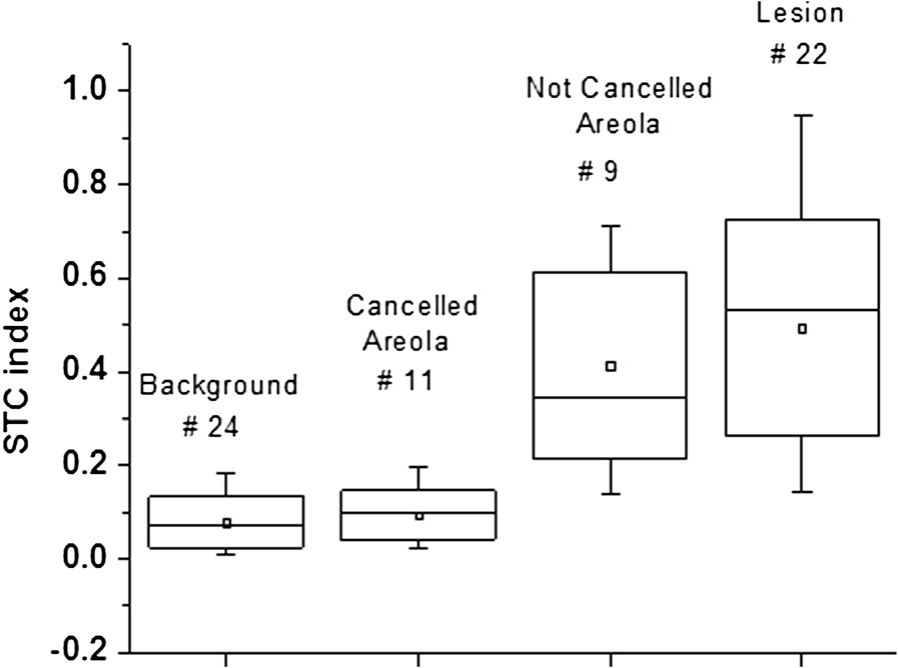
**Box plot of STC index: no contrast in 11 out of 20 areolas.** STC index values in the background normal tissue, STC Cancelled Areola, STC Not Cancelled Areola, and visible tumor. The number of patients for each region of interest is shown above each box. STC, Specific tumor component.

The areola measurements were divided into two sub-categories according to the DOSI enhancement. In the case of a highly attenuated DOSI signal, the areolas are referred to as “Cancelled Areola”; otherwise, the areolas are referred to as “Not Cancelled Areola”. We observed 1 and 11 cases of “Cancelled Areolas” in the TOI and STC index images, respectively. The TOI case of “Cancelled Areola” was found in a peri-menopausal woman with high breast density (BI-RADS 4). High TOI signal is usually seen in the areola complex of similar breast tissue type. For this particular case, we suspect the tumor had exceptionally high metabolic activity that was driving the unusually elevated TOI tumor values relative to normal dense tissue. The tumor had a Tubular Nuclear Mitotic (TNM) score of 9/9, which potentially supports this conjecture. Unfortunately, other specific details of the tumor pathology, such as hormone receptor and HER2 status, or ki67 index were not available for this patient. Figure 5 summarizes the STC index values in background normal tissue, lesion and areola. This plot shows that the STC signal from the “Cancelled Areola” is in the same range as the signal from the background normal tissue. In these cases, the STC index is only sensitive to the tumor and the dense glandular breast tissue of the areola is similar to the background healthy tissue. Since the areolar region contains the densest glandular tissue of the breast, this result suggests that DOSI has the potential to detect tumors in some young and very dense breast where other imaging modalities may fail.

The underlying reason for the cancellation of glandular tissue in the STC index maps of only 11 out of 20 subjects is not yet fully understood. One possibility is that the metabolic activity occurring in the fibroglandular normal tissue of premenopausal women may be involved. Breast parenchyma is known to change throughout the menstrual cycle. This may result in changes for certain chromophores, such as water, in the glandular tissue that could be misclassified as malignant tissue. A modified STC index would be required to compensate for these variations. Another possibility is that the spectral bands used for the calculation of the STC index are not optimal for tumor identification. More work is required to refine the choices of these bands to obtain an index purely unique to malignant tumors. This should improve the STC index capabilities to reliably visualize all tumors in dense breast tissue by fully attenuating the signal from glandular tissue.

While only the TOI and STC index were investigated in this work, other combinations of variables will be explored in future research.

The average maximum dimension of the tumors included in this study was 31 mm (range 12 to 70 mm). It is of importance to be able to also visualize smaller breast cancers. By our protocol criteria, we measure tumors that are at least 10 mm in the greatest dimension. In this study, we investigated these tumors as a proof of the concept that DOSI contrast could be obtained in tumors located in dense breasts. The impact of tumor depth was also not addressed in this work because the DOSI probe incorporated a single imaging view. We expect that some of the results presented in this study may vary depending upon tumor depth and size. However, we suspect that the STC signal is less susceptible to depth dependent variation than the TOI. This is due to the fact that STC contrast is highly sensitive to small differences in normalized spectral features while TOI contrast is based on absolute differences in T/N spectral amplitudes.

## Conclusion

In summary, the high spectral content of DOSI measurements can be used to create complementary contrast functions based on the abundance and disposition of intrinsic tissue chromophores. These features may be useful in tumor detection and diagnosis in difficult to access dense breast regions, such as the areolar complex and other highly vascularized, metabolically active glandular structures.

## Abbreviations

BI-RADS: Breast Imaging Reporting and Data System; ctH2O: Amount of water (%); ctHHb: Concentration of deoxy-hemoglobin (μM); ctO2Hb: Concentration of oxy-hemoglobin (μM); DCIS: Ductal carcinomas *in situ*; DOSI: Diffuse Optical Spectroscopic Imaging; ER: Estrogen receptor; FDPM: Frequency-domain photon migration; HER2: c-erbB2; IDC: Invasive ductal carcinomas; ILC: Invasive lobular carcinomas; L: Left; NA: Not applicable; NAv: Not available; peri: Perimenopausal; PET: Positron emission tomography; post: Postmenopausal; PR: Progesterone receptor; pre: Premenopausal; R: Right; SS: Steady-state (SS); STC: Specific tumor component; TOI: Tissue optical index; T/N: Tumor to normal; TNM: Tubular Nuclear Mitotic score.

## Competing interests

BT, AC, and EG report patents and disclosures, owned by the University of California, which are related to the optical technology and analysis methods described in this work. The other authors do not have competing interests. The diffuse optical imaging instrumentation described was constructed in a university laboratory and this research was completed using federal grant support.

## Authors’ contributions

AL designed the study, acquired the data, carried out the data processing and analysis, and drafted the manuscript. AFD and MC assisted with data acquisition and patients’ accrual. AEC and BJT were involved in the interpretation of results. AEC, EG and BJT conceived of the study and helped to draft the manuscript. BJT participated in the study design and coordination. All authors read and approved the final manuscript.

## Supplementary Material

Additional file 1 Subject and tumor information. Click here for file
