# Supplementary material for: Assessing tumor contrast in radiographically dense breast tissue using Diffuse Optical Spectroscopic Imaging (DOSI)
Source: Breast Cancer Res. 2013 Sep 26;15(5):R89. doi: 10.1186/bcr3485 (PMC3979060; doi:10.1186/bcr3485)
Supplement: Additional file 1 — Subject and tumor information. [file bcr3485-S1.pdf]

Supplementary Material

Supplementary Table: Subject information

Patient	Age (year)	Menopausal status	BI-RADS	Histology	TNM score	Tumor subtype	Side	Max tumor Size (mm)
1	41-45	pre	4	ILC+LCIS	8	ER+, PR+, HER2 -	L	19
2	21-25	pre	4	DCIS	NA	NAv	L	16
3	36-40	pre	3	IDC	9	tripple +	L	21
4	26-30	pre	4	IDC	4	ER+, PR+, HER2-	R	45
5	46-50	pre	4	IDC	4	ER+, PR+, HER2-	L	NAv
6	21-25	pre	4	DCIS	NA	ER+, PR+, HER2 NAv	L	49
7	46-50	peri	4	IDC	Nav	NAv	R	40
8	36-40	pre	3	IDC	4	ER+, PR+, HER2 NAv	L	30
9	36-40	pre	4	IDC	9	ER-, PR-, HER2 NAv	L	50
10	31-35	pre	3	IDC	6	ER-, PR-, HER2+	L	13
11	41-45	pre	4	IDC	5	ER+, PR+, HER2 NAv	R	10
12	46-50	pre	3	IDC	6	triple +	R	21
13	31-35	pre	3	IDC	6	triple +	R	43
14	41-45	pre	4	ILC	Nav	ER+, PR+, HER2-	R	29
15	46-50	pre	4	IDC	6	ER+, PR+, HER2-	R	15
16	41-45	pre	3	ILC	7	ER+, PR+, HER2-	R	40
17	41-45	post	NAv	IDC	7	ER+, PR+, HER2-	R	23
18	36-40	pre	4	ILC	3	ER+, PR+, HER2-	L	60
19	41-45	pre	3	ILC	6	ER+, PR+, HER2 -	R	38
20	41-45	peri	4	IDC	9	triple -	R	52
21	26-30	pre	4	IDC	8	ER+, PR+, HER2-	R	40
22	31-35	pre	4	IDC	8	ER+, PR-, HER2-	R	70
23	41-45	pre	4	IDC	9	triple -	R	12
24	36-40	pre	4	IDC	7	triple +	L	15

TNM – Tubular Nuclear Mitotic score; pre - premenopausal; post - postmenopausal; peri - perimenopausal; IDC – Invasive Ductal Carcinoma; ILC – Invasive Lobular Carcinoma; DCIS – Ductal Carcinoma In Situ; ER – Estrogen Receptor; PR – Progesterone Receptor; HER2 – c-erbB2; L - left; R - right; NA - Not Applicable; NAv - Not Available;
Note that the exact age of the patients is not provided to ensure patient confidentiality.
